# Advances in the Application of Preclinical Models in Photodynamic Therapy for Tumor: A Narrative Review

**DOI:** 10.3390/pharmaceutics15010197

**Published:** 2023-01-05

**Authors:** Rong Yu, Ewetse Paul Maswikiti, Yang Yu, Lei Gao, Chenhui Ma, Huanhuan Ma, Xiaobo Deng, Na Wang, Bofang Wang, Hao Chen

**Affiliations:** 1 The Second Clinical College of Medicine, Lanzhou University, Lanzhou 730030, China; 2 Department of Surgical Oncology, Second Hospital of Lanzhou University, Lanzhou 730030, China; 3 Key Laboratory of Digestive System Tumor of Gansu Province, Second Hospital of Lanzhou University, Lanzhou 730030, China

**Keywords:** photodynamic therapy, preclinical model, organoids, tumor

## Abstract

Photodynamic therapy (PDT) is a non-invasive laser light local treatment that has been utilized in the management of a wide variety of solid tumors. Moreover, the evaluation of efficacy, adverse reactions, the development of new photosensitizers and the latest therapeutic regimens are inseparable from the preliminary exploration in preclinical studies. Therefore, our aim was to better comprehend the characteristics and limitations of these models and to provide a reference for related research. Methods: We searched the databases, including PubMed, Web of Science and Scopus for the past 25 years of original research articles on the feasibility of PDT in tumor treatment based on preclinical experiments and animal models. We provided insights into inclusion and exclusion criteria and ultimately selected 40 articles for data synthesis. Results: After summarizing and comparing the methods and results of these studies, the experimental model selection map was drawn. There are 7 main preclinical models, which are used for different research objectives according to their characteristics. Conclusions: Based on this narrative review, preclinical experimental models are crucial to the development and promotion of PDT for tumors. The traditional animal models have some limitations, and the emergence of organoids may be a promising new insight.

## 1. Introduction

Since the first application and utilization of photodynamic therapy (PDT) in the year 1972 to date, in tumor treatment, it has gained more popularity and has been in the spotlight in the treatment of solid tumors due to its advantages of non-invasive, significant efficacy and good selectivity [1,2,3,4,5,6]. PDT strategy and phenomenon involve the damaging and destruction of target tissues through the interaction of photosensitizers (PS), specific wavelength excitation of light, and oxygen usage involvement [7]. Moreover, one main advantage of PDT in the treatment of tumors is that photosensitizers promptly concentrate in malignant lesions, and less accumulate in normal tissues, thus reducing the off-target toxicity of PDT [8]. The anti-tumor mechanisms of PDT include three aspects; (1) it directly damages and destroys tumor cells by releasing cytotoxic substances, (2) it stimulates the body’s immune response against tumors and (3) its destruction capability to blood vessels leads to tumor cell nutrient deficiency [9]. The well-tolerated PDT and its potential synergistic effect with other therapeutic strategies have attracted wide attention in the field of tumor therapy [10]. However, there are still many challenges in PDT-related strategies, such as how to improve the specificity of photosensitizers, develop new photosensitizers, enhance the efficacy of PDT and broaden its indications. Therefore, in solving and addressing these burdensome problems and challenges, exploration based on preclinical models has been indispensable. Nonetheless, over the past decades, it has become increasingly clear that conclusions drawn from animal studies cannot be effectively construed into human studies. In a study and in findings of more than 60 highly cited animal studies published in top journals from 1980 and 2000, only about a third of the results were rendered to human trials [11]. Collectively, in cancer research, the average rate of successful translation from animal models to clinical cancer trials is less than 8% [12]. Nevertheless, experimental models of photodynamic therapy for various tumors are summarized and elaborated on in this review, aiming to provide a solid and firm basis with reference to preclinical studies of PDT in tumor management. 

## 2. Review Methodology

### 2.1. Literature Search and Selection

In this literature review, a search was conducted in three scientific literature databases (PubMed, Web of Science and Scopus), and our search was limited to articles published in the English literature between 1997 and 2022. For all fields search, the search terms include [cancer, tumor], [chorioallantoic membrane, zebrafish, mouse, rat, rabbit, pig, organoid] and [photodynamic therapy, PDT] for OR searching in each term set, and each term set was used together for AND searching. We used the inclusion and exclusion criteria outlined below as the first step in screening those papers. 

**Inclusion criteria:**

- original research articles about PDT for tumor;
- in vitro and in vivo research;
- detailed information including cell name, animal strain, molding method, fluence, irradiation wavelength, the duration between PS treatment and irradiation and the duration between irradiation and viability assays;
- articles in English.

**Exclusion criteria:**

- literature of non-original research;
- studies containing insufficient data;
- written in a language other than English.

Based on these criteria, we screened the literature according to the titles and abstracts to assess the suitability of the search results. Next, the full text of the selected studies was analyzed to assess whether they met the inclusion criteria. We analyzed the methodological details of inclusion to minimize the risk of bias in individual studies. The whole search process and all research selection were done by two authors. 

### 2.2. Results

At the end of the search, the initial results were 1056 articles, and after screening the titles and abstracts of these papers, 340 relevant articles met the inclusion criteria. Then, the full paper was reviewed, and 40 relevant articles were included in the analysis with detailed methods. In the following section, we summarized the findings of this literature review, focusing on the construction methods of all models, PDT processing parameters and observation indicators and the application scope, advantages and disadvantages of the models.

## 3. Chorioallantoic Membrane (CAM) Model

CAM is a respirable membrane surrounding chicken embryos with an abundant and clear network of blood vessels on its surface, which can be used as an intermediate model between in vitro cell culture and laboratory in vivo animals. CAM is often incorporated in studies involving tissue transplantation, tumor growth and metastasis, drug delivery and toxicological analysis, as well as angiogenesis and anti-angiogenesis molecules and hemodynamics [13,14,15,16,17,18].

Furthermore, CAM is a convenient and feasible model system in the PDT specialties and areas, mainly because its membrane can be obtained and accessible. Moreover, it can be transplanted or rapidly develop tumors on the membrane, which is convenient for studying and the exploration of PDT-induced vascular injury under the microscope in vivo techniques. In addition, CAM is a simple model to operate and is inexpensive. Since CAM is innervated late in embryonic development, the ethical issues are relatively simplified compared to other in vivo models [19]. However, CAM cannot be used in the study of tumor immune microenvironment due to its inherent immune deficiency [20]. This model is generally used to rapidly detect the damaging effect of PDT on the tumor vascular system (Table 1).

In studies involving PDT, although it is relatively simple to visualize tumors and blood vessels growing on CAM, the model is mainly used to study the damage of PDT to normal CAM blood vessels and is less utilized in the evaluation effects of PDT on tumor vessels developing on CAM. Similarly, it is extremely difficult to effectively deliver photosensitizers to tumors. For this reason, Hornung et al. used an intraperitoneal (IP) injection of a chicken embryo as a method of systemic photosensitizer delivery, to avoid intravenous (IV) injection’s time-consuming and low success rate, drug limitations of yolk sac injection and disease types limited by the local injection [22]. In addition, indentation was created on the surface of CAM to enhance cell localization during the transplantation of tumor cells, and the ultimate tumor transplantation success rate reached 80%. Thus, the absorption kinetics of the tumor growing on CAM and the PDT response in the CAM vascular system were evaluated. On the contrary, clinical PDT application usually involves IV administration of photosensitizer (PS), so further research is needed to improve the targeted delivery and binding strength of PS. On the other hand, Plenag et al. incorporated the use of traditional photosensitizer with hydrophobic hypericin and different biocompatible liposomes and injected CAM intravenously to enhance the bioavailability and photodynamic activity [25]. After light exposure, it was found out that the vascular damage was at grade 3–4 (vascular collapse with a diameter of 30–70 mm). A model of PDT targeting tumor vascular damage without affecting the oxygen-dependent PDT effect was successfully established.

## 4. Zebrafish Model

Zebrafish is another non-mammalian animal model that can be incorporated and used in PDT research and investigation. Zebrafish only have a non-specific immunity at an early stage and do not have a well-distinguishable specific immunity, which is used to construct the xenograft cancer model [26]. In addition, researchers have successfully constructed various zebrafish tumor models such as gastric cancer and lung cancer through targeted gene regulation [27,28,29].

Furthermore, they have high fertility and can carry out high-throughput experiments in a relatively short period of time. Meanwhile, zebrafish larva transparency (real-time visualization) can be used in the measurement and studying of genes through the spatiotemporal specificity of gene expression and editing [30,31]. Moreover, zebrafish have similarities in the cardiovascular, nervous and digestive systems as those in mammalian species, and have approximately 75% similar to those in the human genome [32]. The zebrafish model is less costly than the mammalian model. However, the culture temperature of zebrafish is generally lower than 34 °C, which affects the growth of tumor cells. Notably, the immune system of zebrafish fully matures on the 21st day of embryonic development, which often limits, restricts and hinders the experiment period. All in all, only about 25–100 tumor cells can be injected into zebrafish embryos, hindering the long-term transplantation of tumor experiments [33].

In some recent studies on tumor PDT, zebrafish tumor models have been mainly using their embryos to construct tumor models and disease-free models, respectively to verify the effectiveness and safety of PS. On this note, Huang et al. verified the safety of photocatalyst (Ir3) by microinjecting Ir3 into two zebrafish species (wild-type, AB strain; transfected with green fluorescent protein, FLK strain) [34]. Scanning with confocal electron microscopy after light treatment revealed that Ir3 did not interrupt the embryonic development of AB strain, and no obvious damage was found by the green fluorescence signal of FLK strain, indicating that Ir3 was highly biocompatible without dark toxicity. Similarly, Chen et al. applied this model to investigate the cytotoxicity of the phototherapy Nano compounds Ce6-HA-CIS [35]. Moreover, their research group exposed zebrafish embryos to different concentrations of Ce6-HA-CIS. The zebrafish embryo development was monitored and visualized on a microscope equipped with a digital camera. However, embryos treated with different concentrations of Ce6-HA-CIS showed no developmental delay and shared the same phenotypic characteristics as those in the control samples. Notably, the embryo survival rate was recorded to be 95%. Significantly, this showed that zebrafish were an effective biological system in the investigation of the biosafety of nanomaterials. On the other hand, Dib et al. in order to verify the targeted anti-tumor effect of mannose-functionalized porphyrin-based bridged-silsesquioxane nanoparticles (PORBSNs-mannose) activated by two-photon excitation (TPE) in vivo, fed zebrafish embryos in a 28.5 °C tank and injected human breast cancer cells MDA-MB-231 into the periocular space of the fertilized embryos [36]. Strikingly, the larvae were cultured at 32 °C and the zebrafish with xenografts were observed under a microscope. Diluted PORBSNs-mannose was administered intravenously on xenografted zebrafish larvae at 4 days postfertilization. Two days later, the injection group was irradiated with 800 nm two-photon for 1.57 s. Confocal microscopy was used to observe the xenograft tumor volume in the injection group and the control group, and the results showed that the xenograft tumor volume in the injection group was significantly reduced. It further revealed that the zebrafish was a valuable biological system in the investigations of the biological efficacy of nanomaterials.

## 5. Mouse Model

Experimental mice models have become the most commonly used model organisms in replacement of humans in tumor research due to their short growth period, fertility efficacy, adequate physiological characteristics and complete genome sequencing [37]. Experimental tumor models are generally constructed in several ways, such as xenograft, syngeneic, genetic engineering editing and toxicity induction, using different strains of immunocompetent or immunodeficient mice [38].

### 5.1. Xenograft Mouse Model

A xenograft model is currently an effective tool for the study of malignant diseases by establishing human tumors in immunodeficient mice. The establishment of xenograft models usually involves the transplantation of tumor cell lines or tissues from tumor patients [37]. Immunosuppressed mice are usually T cell-deficient thymus nude mice or T/B cell-deficient severe combined immunodeficiency (SCID) mice, and modified SCID, called NOD/SCID, with greater deficiencies in macrophage function, complement-dependent hemolytic activity and NK activity [39]. As the requirements of xenograft models were in more demand, NOG (IL2rg mutated non-functional protein) and NSG mice (IL2rg completely non-expressed) developed from the mutation of IL-2 receptor γ chain (IL-2rgnull) on the basis of NOD/SCID were severely deficient in innate immunity and completely deficient in adaptive immunity. It was used for the transplantation of several more types of tumors, especially those pertaining to hematological malignancies [40,41].

The main tumor source of the xenograft model is the human species, so it can better simulate the original biological characteristics of a tumor [42]. However, due to the increased susceptibility of mice to infection, they must be kept in a specific pathogen-free environment, which has an increment in research costs. Moreover, the grafts must also be free of pathogens, with transplantation operation time limitations and relatively strict aseptic technical requirements. Xenograft mouse models in tumor PDT studies are usually those involving nude or SCID mice transplanted with well-grown human cancer cell lines or fresh tumor tissues. Similarly, transplantation routes usually include subcutaneous (for direct observation of tumor growth), in-situ (for simulating the source environment of tumor growth) and intraperitoneal transplantation (for monitoring tumor spreading) (Table 2).

On the contrary, Cheng et al. demonstrated that tissue factor (TF) expression was higher in human lung cancer cells and vascular endothelium of transplanted tumors in nude mice than in normal cells and blood vessels [45]. Strikingly, in order to apply this finding to the clinical treatment of PDT-targeted anti-lung cancer, they conducted the first in vivo dose-efficacy and safety experiment with a photosensitor (VII/NLS-SnCe6) chimeric with mouse factor VII protein, a natural ligand of TF. The results showed that the tumor load was effectively inhibited in the VII/NLS-SnCe6 PDT group compared to the control group. Furthermore, post-screening VII/ LS-SNCE6 concentration, light parameters and treatment frequency, a second in vivo experiment was conducted to optimize the treatment effect, indicating that the tumor was significantly inhibited in the experimental group, and 20% of the mice had tumor eradication without recurrence. Compared with subcutaneous transplantation, studies have pointed out and demonstrated that tumor growth in orthotopic transplantation is faster and tumor vascular density is higher with tumor cells coping and striving better with a low degree of hypoxia, which contributes to a better photodynamic therapeutic effect with increased clinical similarity [50]. Contrarily, Grossman et al. established a pleural metastasis model of lung cancer to validate the feasibility and efficacy of PDT induced by methyl pyropheophorbiide derivatives (2-[1-hexyloxyethyl] 2-devinyl Pyropheophorbiide-a, HPPH) (in a Phase I clinical trial) in disseminated non-small cell lung cancer [46]. Magnetic Resonance Imaging (MRI) scanning and calculation of tumor growth showed that the tumor growth in the light group was significantly lower than that in the control group (*p* < 0.04). This provides guidance for the establishment of a preclinical research model of PDT for the treatment of diffuse thoracic diseases. Pancreatic cancer cells MIA-PaCa-2 can form an anoxic tumor model, which has been previously used in the therapeutic efficacy experiments of hypoxic-activating drugs [51]. However, Sheng et al. prepared pH-sensitive polymer-coated CaO_2_ nanoparticles, which could produce oxygen under the stimulation of a low pH value in solid tumors [49]. Prior to PDT, the tumor pO_2_ of xenograft pancreatic cancer mice was increased by 6.5 mmHg on average within 10–30 min after oral administration of the nanoparticles, which significantly improved PDT-mediated efficacy (*p* < 0.001). These studies indicate that xenografted mouse models play an essential role in the research of novel composite nano photosensitizers for tumors.

### 5.2. Syngeneic Mouse Model

The syngeneic mouse model, also known as the homogenic mouse model, involves inbred mice inoculated with tumor cell lines of the same origin and background. The recipient mice have a complete murine-derived immune system, which is compatible with allograft tumor tissue and can maximize the simulation of the tumor microenvironment [52]. Therefore, it is used to evaluate the interaction between tumor cells and immune cells and the efficacy of tumor immunotherapy [53,54].

Currently, there are several mouse homogenic tumor cell lines of various tumor types, which are used for preclinical in vivo verification, but their application in research exploration is limited by the following factors; (1) most of these mouse tumor cell lines are derived from carcinogenic-induced models, which carry complex and unstable genetic changes; (2) most cell lines grow rapidly in vivo and, therefore, allow only a short period of treatment for investigation prior to the tumor size reaching the Institutional Animal Care and Use Committee (IACUC) limitations. Neither of these can simulate the situation of human tumors [55]. However, in recent years, due to the need for preclinical models of tumor immunity, the application of such models has been on the rise. At present, among more than 400 inbred mouse strains, BALB/c, C57BL/6(black) and C3H are the most commonly used in PDT anti-tumor studies (Table 3).

PDT is an option for local treatment of other unresectable tumors and induces systemicanti-tumor immunity [62]. There are some uncertainties as to whether this local treatment will completely eliminate all the tumor cells. In regard to this, Kammerer et al. analyzed the transcriptome changes of prostate cancer mice before and after receiving PDT by oligonucleotide microarray and found out that the protein encoded by the high-dose up-regulated gene after PDT belonged to the cell stress pathway or cell cycle arrest, and the immune gene of tumor cells was up-regulated post non-lethal PDT dose irradiation [56]. These included and were not limited to the chemokine genes CXCL2, CXCL3 and IL8/CXCL8, as well as the IL6 and its receptor, IL6R, which could promote inflammatory responses and also promote tumor growth. It was suggested and demonstrated that the therapeutic effect could be improved by controlling the PDT dose to avoid the tumor stimulation pathway. In order to achieve complete anti-tumor immunity, studies have suggested and found out that the combination of immunogenic cell death (ICD) and antigen-presenting cells (APCs) phagocytosis enhancement to induce intrinsic cancer vaccines was possible [63]. Furthermore, Kim et al. proposed whether the combination of PDT and special PS could induce ICD in tumor cells and improve the efficacy of tumor immunotherapy [59]. Their research group injected Ce6-embedded Nano photosensitizing agent (FIC) into the tumor of syngeneic melanoma mice to evaluate the antitumor efficacy and immune response of PDT alone and combined with Rho-kinase (ROCK) inhibitor, which was combined with anti-PD-L1 antibody. Collectively, the results revealed that the combination of local FIC-PDT and a ROCK inhibitor exerted a cancer vaccine-like function, stimulating antigen presentation and initiating tumor-specific cytotoxic T cells, and sensitizing PD-1/PD-L1 immune checkpoint blockade to evoke and trigger systemic antitumor immunity. On the contrary, Xie et al. established a bilateral colon cancer mouse model of allotransplantation and verified that PDT targeting mitochondrial receptor 18 kDa translocation protein (TSPO) in the treatment of colon cancer stimulated ICD and gave rise to a distant effect [60]. At the end of a 14-day period of observation, the number of primary tumors treated with TSPO-PDT was significantly smaller than that in untreated controls (*p* < 0.001), and the contralateral tumor growth was significantly inhibited in the treatment group (*p* < 0.0001). The above studies indicated that the syngeneic mouse model was an effective model in tumor PDT activation in vivo immunity.

### 5.3. Genetically Engineered Mouse Models (GEMMs)

GEMMs have since been constructed to more closely mimic the progression of human diseases in a controllable way [64]. There are several types of GEMMs, including transgenic, knock in and knockout mouse models [65], which are mainly used for preclinical studies of candidate excitation genes, metabolic mechanisms and novel therapies [66].

GEMMs tumors form in a microenvironment within a conducive favorable natural immunity. On the other hand, advanced tumors have very similar histopathological and molecular characteristics to human tumors, exhibit genetic heterogeneity and can spontaneously metastasize [67]. Nevertheless, GEMMs construction requires high technical requirements, long modeling time and high cost. The inherent defects of the model also limit its wide application in PDT’s tumor research (Table 4).

The transformation of tumor PDT research has been hindered; that is, some tumor cell lines and tissues have been sensitive to PDT after transplantation into experimental animals while spontaneous tumors in human patients have not been responding well to PDT. To date, Córdoba et al. used MT-ret mice (transgenic melanoma mice) to simulate the clinical situation and disease presentation [69]. Post-screening the optimal PS dose and light parameters from human and murine melanoma cells in vitro, PDT treated transgenic melanoma mice, and the results showed that the treatment did not significantly alleviate tumor progression. In contrast, pancreatic cancer with poor survival thorny prognosis has achieved better results in vivo experiments of PDT in transgenic mouse models. Abd-Elgaliel et al. hybridized LSL-KRasG12D mice, floxed-p53 mice and pancreatic-specific Pdx-1-Cre mice to produce KRasG12D mice with opportunistic p53 deletion and endogenous mutation [68]. Littermates without pancreatic ductal adenocarcinoma (PDAC) served as controls. The photosensitizer 5-ALA precursor was injected through the tail vein and the pancreas was irradiated with light. The apoptotic process of the tumor tissue was evaluated 1-day post-treatment. Interestingly, the results showed that the 5-ALA precursor was activated in Cath E (a proteolytic enzyme highly expressed in PDAC) positive tumors, resulting in the apoptosis of cancer cells while apoptosis was absent in the pancreatic tissues of control mice. It was suggested that Cath E expressed mice were the dominant target group for PDT treatment of PDAC. These studies indicated that GEMMs are effective biological system for selecting the dominant population for PDT intervention.

## 6. Rat Model

Compared with mice, rats are much larger in size and easier to operate upon, and their physiological processes and anatomical structures are much simpler to locate, which can be utilized for the evaluation of the cardiovascular system, metabolic system disorders, and the nervous system [71]. In addition to the aforementioned systems, hepatocellular carcinomas, breast cancers and other tumors can be analyzed and investigated upon in these models. Collectively, the rat model was used in multiple studies of PDT to verify the reduction of anti-tumor adverse reactions of PDT and the effectiveness of PDT combined with anti-tumor strategy (Table 5).

Studies have proved that metformin can increase the effectiveness of anti-cancer treatment in oncology patients with reduced insulin levels [77]. Furthermore, Nenu et al. proposed whether the combination of this anti-diabetic drug and PDT could increase the therapeutic effect, and Wistar rats were selected as the group experimental models [72]. This was in regard to the fact that Walker 256 tumor cells were easily transplanted into rats and obese rats with Walker 256 tumor had lower insulin circulation than tumor-free obese rats. The results showed that the phototoxicity of PDT mediated by ROS production and increased lipid peroxidation was amplified and the apoptosis index of tumor cells was higher in the combination treatment group. In order to verify the safety and effectiveness of PDT combined with surgery in the treatment of breast cancer, Ferreira et al. constructed 12 chemical induced breast cancer rats (sensitive to carcinogen induction) models [74]. It was categorized into the control group (G1), PDT treatment group (G2), surgical resection group (G3) and PDT treatment group immediately after surgical resection (G4). The results revealed that the tumor growth had been delayed in the PDT group, there was no tumor recurrence in the G4 group within 12 weeks after chemical induction and the recurrence rate in the G3 group was 60% after 12 weeks of chemical induction. It has been suggested that this combination of therapy could destroy residual tumors and prevent recurrence. Contrarily, Gries et al. established 42 orthotopic xenograft model of glioblastoma (GBM) rat models to verify whether transmembrane receptor neuroproteinase-1 (NRP-1) is a relevant molecular target to promote the anti-vascular effect of photodynamic therapy (VTP) [76]. They combined NRP-1-targeted KDKPPR peptide and PS with nanoparticle AGuIX(in Phase II clinical trials for the treatment of brain metastases with radiotherapy). Model groups were treated with PDT under the guidance of MRI. Cranial window models and parametric maps obtained from T2-weighted MRI showed the prolonged retention of human xenograft GBM in the vascular system. The absence of nanoparticles in the brains of tumor-free animals was checked. Follow-up post-VTP showed delayed tumor growth and decreased metabolism. Therefore, the rat models show advantages in the study and evaluation of PDT therapy combined with clinical intervention.

## 7. Rabbit Model

The thick skin and subcutaneous lipid layer of rabbits mimic the depth of human tumors to a larger extent. It has been previously reported that the rabbit squamous cell carcinoma (VX2) model exhibits very similar tumor characteristics to those often encountered in human. Moreover, VX2 cancer ailment can be implanted into multiple rabbit tissues and has significant similarities to human in situ tumors in terms of vascularization, histology, and biological features [78]. In situ neoplasms in rabbits appear to be more similar to human clinical neoplasms compared to those in mouse models [79].

The rapid development of tumors in rabbits and the high recurrence rate hinder the evaluation of PDT’s effect in large animal models. In regard to solving this problem, researchers have upgraded the reliability of their results in conjunction with a mouse tumor model. Table 6 reflects the application of rabbit models in recent tumor PDT studies.

As mentioned earlier, the anti-tumor strategies of PDT mediated by nanomaterials have been extensively studied. Furthermore, they are mainly limited to the treatment of mouse tumor models, and there are few clinically relevant studies in large animal models in this specialty and area. However, Liu et al. established 12 rabbit VX2 breast xenograft models and 20 subcutaneous xenograft tumor models in nude mice [84]. First, the anti-tumor efficacy and biosafety of PDT mediated by macrophage-loaded photoactive substances were monitored in mouse models. The ablation process was then monitored by two-dimensional ultrasound, scratch-elastic imaging (SWE) and contrast enhanced ultrasound (CEUS) in rabbit models that were closer to the human tumor microenvironment. The results showed that compared with the control group, the temperature of the tumor area in the treatment group rapidly increased to 65 ℃, the laser penetration distance was 10 mm, and the tumor was completely cured. This provided a new strategy for the treatment and monitoring of tumors in large animal models. In a study conducted by Muhanna et al., the rabbit VX2 thyroid tumor models related to human anatomy and human papillary thyroid carcinoma mouse models related to biology were constructed, and the minimally invasive and specific treatment of thyroid cancer by PDT mediated by the porphyrin-HDL nanoparticle (PLP) was evaluated [83]. The intrinsic fluorescence tracking of PLP showed that the tumor accumulated preferentially than the surrounding tissue. This result was consistence with the subsequent rabbit tumor models in vivo experiments. Further observation of the tumor and the peripheral recurrent laryngeal nerve (RLN) by continuous section microscope showed that PLP accumulated significantly in the tumor but did not accumulate in the nerve tissue. The survival follow-up showed that PLP-PDT could completely ablate the tumor tissue. Similarly, in order to simulate deep tumors, Chen et al. demonstrated the effect of X-ray-induced photodynamic therapy (X-PDT) under the action of a new sensitizing agent, copper-cysteamine at the animal level and established a subcutaneous mouse model of breast cancer and a rabbit model of VX2 hepatocellular carcinoma in situ [82]. These outcomes had some significant differences between the X-PDT group and the control group in tumor cell migration and the cell proliferation antigen. Collectively, an MRI evaluation showed that X-PDT inhibited the growth of subcutaneous tumors in mice and deep tumors in rabbits (*p* < 0.05) with no obvious in vivo toxicity. Therefore, the rabbit models have absolute advantages in simulating deep tissue tumors and evaluating the therapeutic efficacy and specificity of PDT.

## 8. Pig Model

Pigs are similar to humans in anatomy, physiology, nutritional habits and other aspects. In particular, pigs and humans have a similar skin tissue structure, as well as a similar epithelial repair and reproduction, subcutaneous fat layer, endocrine functions and metabolic processes [85]. Unlike small animals, the same catheters and equipment can be used for local treatment in pig models as in humans. In preclinical studies of PDT treatment for tumors, local tissues or organs of pigs are often used to simulate the human environment. It is not simple and user-friendly to establish a tumor model in pigs, so they are rarely used in tumor PDT research, and it is generally constructed as a light dose model to verify the safety and effectiveness for new PDT therapy.

Murray et al. in order to investigate the feasibility and safety of WST11 vascular targeted photodynamic therapy (VTP) as a non-surgical alternative to uroepithelial cell carcinoma, a preclinical porcine model with appropriate tissue sources in the lumen of normal organs was established [86]. They administered WST11-VTP (50–200 mW/cm^2^, 10 min, 30–120 J/cm^2^) to tumor-free Yorkshire pigs under anesthesia via a ureteroscope. Furthermore, enhanced CT scans were performed on bilateral kidneys and ureters at 24 h, 1, 2 and 4 weeks post-treatment, and HE staining was performed on organ tissues. The results showed that the ureter developed superficial necrosis at 24 h post 50 mW/cm^2^ light exposure, superficial urothelial regenerated at the treatment site at 4 weeks. No hydronephrosis was found on CT within 4 weeks. Notably, the ureter at a light dose of 200 mW/cm^2^ could produce deep necrosis through the muscularis propria or serous membrane. Doeveren et al. planned to establish a light dosimetry model to deal with the “scattering effect” [87,88] caused by PDT in the treatment of luminal lesions [89]. According to the CT image data and the optical measurement before PDT treatment, in the case of scattering effect, laser flux rate on the complex sinus lumen surface was calculated firstly. The porcine tissue phantom consists of a 3D printed shell, cloaked inside with an average 15 mm thick homogeneous layer of porcine muscle tissue sutured and secured to the rigid mesh structure, thus creating a tissue cavity that mimics sinonasal geometry. Then, 8 sets of diffuse reflectance were measured on the pig tissue model, and the surface flux rate distribution of all possible light source locations on this model was calculated to find the optimal light source location. In the process of PDT, the target area was effectively illuminated in this location, and the light dose around the target area was reduced to the minimum dose possible. In addition, in the PDT irradiation of skin tumors, the low local effective absorption rate of photosensitizers has always been a challenge that researchers are aspiring to improve and have some significant breakthroughs. Therefore, Reis et al. developed the inclusion complex of hydroxypropyl-β-cyclodextrin (HP-βCD) and aluminum-chloride phthalocyanine (AlClPc) mediated by iontophoresis, which increased the solubility of photosensitizers and maintained the production capacity of the reactive oxygen species (ROS) [90]. Using pig ear skin as an experimental model and irradiated under different photodynamic conditions, the analysis showed that AlClPc skin penetration increased by 2.3 times in a shorter time. It was found and suggested that iontophoresis photosensitizers may be an effective and noninvasive local treatment for skin tumors. Therefore, porcine models can be used as the first choice to evaluate the efficacy of PDT in the study of tumors with lacunar structure requiring medical intervention.

## 9. Organoid Model

Organoids are three-dimensional (3D) self-organizing structures grown from stem cells, which to some extent express many characteristics of derived organs or tissues [91,92]. Moreover, organoid cultures can be derived from tumor tissues or normal cells of tumor patients and exhibit cancer heterogeneity, including morphological changes, gene expression, and the ability to replicate parental tumors [93,94]. Tumor organoid models have become essential preclinical model systems in cancer research, bridging the gap between high-correlation but low-throughput live animal models and high-throughput but low-clinical relevance two-dimensional cell cultures, which has led to their application in high-throughput drug screening, immune response and other studies [95].

The application of the 3D tumor culture model paves a new path for the treatment mechanism of photodynamic therapy at the mesoscopic scale, especially since microtumors growing on extracellular matrix scaffolds can provide reliable statistical data on the effectiveness of photosensitizers and photodynamic therapies by utilizing high-throughput image-based analyses. Despite the abundant and sufficient information, the use of this 3D culture is not widely used due to the high cost and batch-to-batch variability of establishing the extracellular matrix scaffolds necessary for this culture [96].

In traditional two-dimensional cell culture in vitro experiments, monolayer cells lack tumor heterogeneity due to contact inhibition and uniform oxygen and nutrient supply environment, which affects the evaluation of the efficacy of PDT in tumor treatment. Therefore, Nath et al. constructed a 3D model of ovarian cancer to investigate the effects of PS uptake and PDT under different oxygen gradients [97]. The advantage of this model is that fluorescent protein is used to label and tag cancer cell genes as an indicator of cell viability, and PDT has proven to have a good killing and destruction effect on cancer cells. In addition to tumor organoid models, normal tissues can also be validated by 3D culture as preclinical models. The cholangiocarcinoma (CCA) organoids and monolayer structures of non-tumor organoids established by Fujiwara et al. was contrasted [98]. CCA organoids demonstrated a remarkably high photodynamic activity based on higher accumulation of protoporphyrin IX as a metabolite of 5-ALA compared to non-tumor organoids (40–71% vs. < 4%, respectively), which suggested that 5-ALA-based photodynamic activity had some diagnostic potential for the discrimination of CCA from non-tumor tissues. With variation from other tumor metastasis models in vivo, the organoid model of tumor metastasis in 3D culture is more direct to construct and can reflect the heterogeneity of tumor growth. Broekgaarden et al. cultured the tumor organoid with metastatic human pancreatic cancer cells AsPC-1 on the solidated Matrigel scaffold according to the established 3D adherence culture scheme, ingrained the in vitro micro-metastatic pancreatic cancer model and investigated on the potential of a combined treatment consisting of PDT and subsequent oxaliplatin chemotherapy [99]. The outcomes demonstrated that neoadjuvant PDT enhanced the immediate and prolonged efficacy of oxaliplatin in metastatic pancreatic organoid carcinoma. It follows that organoid model with its unique characteristics would certainly play a huge role in the investigations of PDT treatment of tumors.

## 10. Outlook and Perspectives

**Figure 1:**
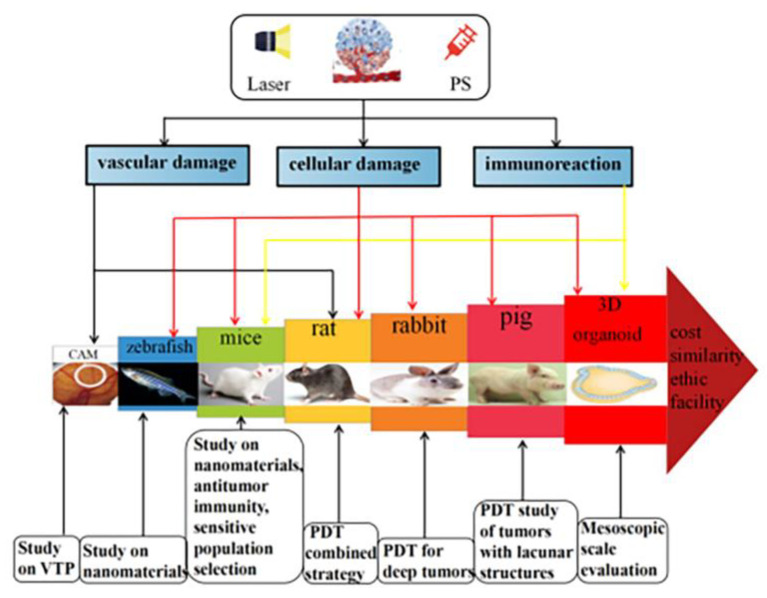
Selection of preclinical models commonly used in tumor treatment by PDT irradiation.

Photodynamic therapy (PDT) is a complex light laser treatment modality where a large array of factors can influence therapeutic outcome. Vascularization, vessel permeability, oxygenation and light distribution in the tissue as well as immune response play a key role in the photodynamic process. The experimental model provides a preclinical evaluation platform for the study of PDT tumor management, which is convenient to observe the effect and mechanism of new photosensitizers and PDT combined treatment strategies, and to monitor the changes in tumor morphology. It gives rise to some promising new insights into the treatment of many types of cancer while each of these factors influencing photodynamic response is in turn influenced by the choice of preclinical model. Suitable models have to be selected according to different research objectives and aims. We have therefore summarized the characteristics and application scope of various models (Table 7), and provided the selection guidance (Figure 1). There are still some problems and challenges in this area; for instance, (1) it is necessary to continue to explore animal model that can simulate the human tumor environment and tumor development to a great extent, and (2) the difference between the PDT model validation results and clinical efficacy still needs to be evaluated for transformation processes. Therefore, it is extremely essential to select a suitable and appropriate animal model to improve the conversion rate in tumor PDT studies. Nevertheless, in vivo animal studies are time consuming, which may also show physiological discrepancies between animals and humans. In recent years, 3D organoids culture technology could closely reflect the pathophysiological characteristics of natural tumorigenesis and metastasis and collaborate with other technologies (organon-on-a-chip, 3D bioprinting and CRISPR-Cas9-mediated homologous unrelated organoid transgene) to overcome the limitations of traditional models and promote the development of more clinical model systems. It is hoped with great anticipation that more studies will explore and come up with the best in vivo experimental model to optimize, simplify and promote the clinical application of PDT so as to benefit more patients with cancer.

## Figures and Tables

**Table 1:** Application of CAM in antitumor study of PDT.

Cancer Type	Cell Line/Graft	PDT Regimen	Indicators	Ref.
Ovarian cancer	tumor tissue	methylene blue liposome, DLI 1 h, 647 nm, 100 J/cm^2^	Chick embryo viability and CAM vascular changes	[21]
Ovarian cancer	NuTu-19 cells (5 × 10^6^ cells, monitor 4 days)	ALA/BPD-MA/ Lu-Tex, DLI 90 min, 635 nm/690 nm/740 nm, 100 mW/cm^2^, 5 J/cm^2^, 10 J/cm^2^, 20 J/cm^2^	The degree of vasculature damage	[22]
Melanoma	C8161 (tumor cell spheroids of 1 mm diameter, monitor 10 days)	protoporphyrin IX, DLI 2.5 h, 532 nm, 4 mJ/cm^2^ per pulse	The degree of vasculature damage, the relationship between the vessel damage and PDT dose	[23]
Mammary carcinoma	EMT6 (7 × 10^6^ cells, monitor 4 days)	2 (mTHPC) liposomal formulations, 650 nm, 3 J/cm^2^	Vascular damage in healthy CAM and cellular damage in tumor xenografted on CAM	[24]
Ovarian cancer	SK-OV-3	liposomes containing hypericin, DLI 7 min, 589 nm, 11.4 J/cm^2^	The degree of vasculature damage	[25]

**Table 2:** Application of xenograft mouse in antitumor study of PDT.

Cancer Type	Cell Line	Animal Model	Methods	PDT Regimen	Indicators	Ref.
Pancreas cancer	AsPC-1/Panc-1	SCID mice	Orthotopic (50 um quantity of the cell-Matrigel solution, monitor 2 weeks)	Verteporfin, DLI 1 h, 690 nm, 40 J/cm^2^	The growth rate and vascular pattern of the tumor	[43]
ESCC	Eca109	Nude mice	Subcutaneous (5 × 10^6^ cells, monitor formation volume of 100–300 mm^3^)	HPPH/ Photofrin, DLI 24 h, 665 nm/630 nm, 135 J/cm^2^	The tumor volume, the weight inhibition value, tumor histology, the weight and mortality rate of mice	[44]
Lung cancer	H460/A549	Nude mice	Subcutaneous (1 × 10^6^ cells/2 × 10^6^ cells, monitor formation volume of 100–300 mm^3^)	Factor VII-targeted Sn(IV)chlorin e6 conjugate, 635 nm, 72 J/cm^2^	The tumor volume, tumor HE staining of the mice	[45]
Lung cancer	H460	Nude mice	Orthotopic (1 × 10^6^ cells, monitor 12 days)	Photochlor HPPH, DLI 24 h, 661 nm, 200 J/cm^2^	The tumor volume and weight of the mice by using MR images	[46]
Mammary carcinoma	MDA-MB 231	Nude mice	Subcutaneous (1 × 10^6^ cells, monitor formation volume of 100 mm^3^)	Phthalocyanine, AlOH-PC, DLI 10 min, 635 nm, 100 J/cm^2^	The tumor growth inhibition	[47]
ESCC	Eca109/Ec9706	Nude mice	Subcutaneous (5 × 10^6^ cells, monitor formation volume of 200 mm^3^)	5-ALA, 630 nm, 400 mW/cm^2^, 96 J/cm^2^	The tumor volume, LDH and ELISA analysis of serum, immunohistochemical staining of tumors	[48]
Pancreatic cancer	MIA PaCa-2	SCID mice	Subcutaneous (5 × 10^6^ cells, monitor 5 weeks)	polymer coated CaO_2_, 205 J/cm^2^, 3 × 3 min	The volume, pO_2_ levels of tumor	[49]

**Table 3:** Application of syngeneic mouse in antitumor study of PDT.

Cancer Type	Cell Line	Animal Model	Methods	PDT Regimen	Indicators	Ref.
Prostate cancer	TRAMP-C2	C57BL/6 mice	Subcutaneous (1 × 10^6^ cells, monitor formation volume of 100–300 mm^3^)	5-ALA, DLI 72 h, 635 nm, 100 J/cm^2^	The expression of genes in tumors proinflammatory	[56]
Glioma/brain	9L.E29	athymic mice	Subcutaneous (1 × 10^6^ cells, monitor 21 days)	Phthalocyanine, EGFpep-Au NP-Pc 4, DLI 4 h, 672 nm, 50 J/cm^2^	The tumor size, the image of the mice, Histology studies and HE Staining of tissue	[57]
Breast cancer	4T1	BALB/c mice	Subcutaneous (1 × 10^6^ cells, monitor 5–7 days)	Photofrin, DVDMS, DLI 24 h, 635 nm, 100 J/cm^2^, 416.7 mW/cm^2^	The tumor volume inhibition ratio, the survival of the mice. Immunohistochemistry and HE staining of tumors	[58]
Uveal melanoma	B16F10	C57BL/6	orthotopic (1 × 10^4^ cells, monitor 10 days)	FIC, DLI 1 h, 655 nm, 300 mW/cm^2^, 180 J/cm^2^	The tumor volume, immunofluorescence assays, flow cytometric analysis of tumor	[59]
Colorectal cancer	MC38	C57BL/6	Subcutaneous (5 × 10^6^ cells, monitor 7 days)	IR700DX-6T, DLI 2 h, 690 nm, 18 J/cm^2^	The tumor volume, DCs and Tregs isolated from the spleens of tumor-bearing mice	[60]
Sarcoma	LM8	C3H	Subcutaneous (2 × 10^6^ cells, monitor formation volume of 90–100 mm^3^)	HMME, DLI 4.25 h, 630 nm, 120 J/cm^2^	The sizes and weights, HE Staining and IHC of tumors	[61]

**Table 4:** Application of GEMMs in antitumor study of PDT.

Cancer Type	GEMMMs	PDT Regimen	Indicators	Ref.
Pancreatic cancer	LSL-KRasG12D-p53-floxed-Pdx-1-Cre	Cathepsin E-activatable 5-ALA, DLI 1 h, 652 nm, 10 J/cm^2^	The TUNEL assay of tumor tissues	[68]
Melanoma	MT-ret transgenic 304/B6	5-ALA, DLI 3 h, 630 nm, 200 J/cm^2^	The tumor size, Histopathological analysis of tumor tissue	[69]
Breast cancer	FVB/NTgN(WapHRAS)69LlnYSJL	Foscan-PEG, SC102, 40 J/cm^2^	Immunohistochemistry, HE staining or multiparameter flow cytometry of tumor tissue	[70]

**Table 5:** Application of rats in antitumor study of PDT.

Cancer Type	Cell Line	Animal Model	Methods	PDT Regimen	Indicators	Ref.
Carcinosarcoma	Walker 256	Wistar rats	Subcutaneous (tumor fragments, monitor formation volume of 1 cm^3^)	Porphyrin TSPP, DLI 24 h, 685 nm, 50 J/cm^2^	Histopathological and immunohistochemical examination of tumors	[72]
Bladder cancer	AY-27 cells	Fischer F344 rats	Orthotopic (5 × 10^6^ cells, monitor 5 days)	ALA, DLI 2 h, 514 nm, 20 J/cm^2^	The cardiac and respiratory rhythm of rats, immunohistochemistry examination of tumors	[73]
Breast cancer	/	Sprague–Dawley rats	Autochthonous (DMBA,5 0 mg/kg)	haematoporphyrin, DLI 24 h, LED 635 nm, 200 J/cm^2^	The tumor size	[74]
Glioblastoma	U87	Athymic Fox1 rnu/rnu rats	Orthotopic (5 × 10^6^ cells, monitor 2 weeks)	5-ALA, DLI 5 h, 635 nm, 26 J	MRI, tumor volume	[75]
Glioblastoma	U87	RH-Foxn1rnu rats	Orthotopic (5 × 10^4^ cells, monitor 10–14 days)	AGuIX-PS, DLI 1 h, 652 nm, 26 J	MRI analysis, 18F-FDG-PET acquisitions, tumor and healthy brain tissue activities	[76]

**Table 6:** Application of rabbits in antitumor study of PDT.

Cancer Type	Cell Line	Animal Model	Methods	PDT Regimen	Indicators	Ref.
Head and neck cancer	VX2	New Zealand white rabbits	Orthotopic (1 mm^3^ fragments, monitor formation longest diameter of 2–3 cm)	Photofrin, DLI 24 h, 630 nm, 60–150 mW/cm^2^, 540 J/cm^2^	The tumor volume, CT scans of tumor	[80]
Retinoblastoma	WERI-Rb	pigmented rabbits	Orthotopic (1.5 × 10^6^ cells, monitor 7 weeks)	verteporfin, DLI 16–95 min, 690 nm, 40–150 mW/cm^2^, 1–3 min, 2.4–27 J/cm^2^	Fundus photography histopathologic examination of retinal	[81]
Hepatocarcinoma	VX2	New Zealand white rabbits	orthotopic (0.5–1 mm diameter fragments, monitor 3 days)	Cu-Cy NPs, DLI 30 min, 6 MV, 100 MU/min for 2 Gy	Body weight of rabbits, MRI system and ultrasonic machine examination of tumor	[82]
Thyroid cancer	VX2	New Zealand white rabbits	Orthotopic (2 × 10^6^ cells, monitor formation diameter of 1 cm)	PLP, DLI 24 h, 671 nm, 100 J/cm^2^	CT imaging of tumor and ymph node, survival study of rabbits, fiber optic laryngoscopy of vocal cord movements	[83]
Breast cancer	VX2	New Zealand white rabbit	orthotopic (1 mm^3^ fragments, monitor 10 days)	NbC@M, DLI 8 h, 808 nm, 2 W/cm^2^	The tumor volume, histopathologic analysis of major organs	[84]

**Table 7:** Comparison of various models in antitumor studies of PDT.

Animal Models	Advantages	Disadvantages	Application
CAM	Short experimental period (several days) Inexpensive Innate immune deficiency In vivo imaging can be performed	limitation to topical sensitization Cancer-immune cell interactions could not be examined	The effect of damaging tumor vasculature
Zebrafish	high fecundity Dynamic visualization of tumor growth in vivo Gene expression can be controlled High throughput and medicine analysis	Zebrafish needs a preferred temperature around 28 °C, which affects the growth of tumor cells Short observation period	Safety and effectiveness of PS
Mouse	Longer observation period (weeks to months) Well-defined genetic background Thorough biological and physiological information High fecundity	Insufficient tumor depth	The effect of damaging tumor cells Association of PDT with tumor immunotherapy response Efficacy of PDT in spontaneous tumors
Rat	The operation is simple Biological property is easy to study	Induced mutations are inherited instably	The effect of damaging tumor cells and vasculature Efficacy of PDT combined anti-tumor strategy
Rabbit	Tumors in situ appear to be more similar to human clinical tumors Thick subcutaneous lipid layers can largely mimic human tumor depth	The tumor develops rapidly The recurrence rate is high	Efficacy and safety of PDT in deep tumors
Pig	Highly homologous to humans The metabolism of skin organs and tissues is similar to that of humans	Few tumor models High feeding cost	Exploration of clinical treatment parameters and device types of PDT
3D organoid	Distinct organ or tissue cancer heterogeneity Mesoscopic evaluation	High cost of cultivation	The affinity of PS for tumor cells

## Data Availability

Not applicable.
